# Role of Individual Motivations and Privacy Concerns in the Adoption of German Electronic Patient Record Apps—A Mixed-Methods Study

**DOI:** 10.3390/ijerph18189553

**Published:** 2021-09-10

**Authors:** Richard Henkenjohann

**Affiliations:** 1 Faculty of Linguistics and Information Science, University of Hildesheim, 31141 Hildesheim, Germany; richard.henkenjohann@hpi.de; Tel.: +49-331-5509-3408; 2 Digital Health Center, Hasso Plattner Institute for Digital Engineering gGmbH, University of Potsdam, 14482 Potsdam, Germany

**Keywords:** personal electronic health records, technology adoption, endogenous motivations, health information privacy concern, mixed-methods, ePA

## Abstract

Germany’s electronic patient record (“ePA”) launched in 2021 with several attempts and years of delay. The development of such a large-scale project is a complex task, and so is its adoption. Individual attitudes towards an electronic health record are crucial, as individuals can reject opting-in to it and making any national efforts unachievable. Although the integration of an electronic health record serves potential benefits, it also constitutes risks for an individual’s privacy. With a mixed-methods study design, this work provides evidence that different types of motivations and contextual privacy antecedents affect usage intentions towards the ePA. Most significantly, individual motivations stemming from feelings of volition or external mandates positively affect ePA adoption, although internal incentives are more powerful.

## 1. Introduction

Providing efficient healthcare has a genuine impact on society, as it directly influences people’s well-being. The use of information technology (IT), and more specifically, electronic health records (EHRs), improves provisioned quality and reduces healthcare costs in general [1]. Healthcare is information-intensive, since many activities are enabled through storing, processing, and analyzing data. An EHR increases efficiency in healthcare delivery, simplifies monitoring patient health, facilitates monetary savings, reduces paper-based errors, and improves diagnoses and treatments [2,3,4,5,6]. Governments and healthcare systems promote national patient health records as “a way of preserving patients’ health and medical information and maintaining their data in a central facility that ideally can be shared between different healthcare providers” ([7], p. 1). An EHR offers “efficiencies in collecting and storing patient information, contributing to continuity of care and alleviating problems such as misdiagnosis or prescription errors” ([7], p. 1). Patient-administered health records, often referred to as personal health records (PHRs), aim to improve this continuity of care while simultaneously realizing the right to informational self-determination in that each patient is made the owner of all disease-related data [8]. In a PHR, citizens can add valuable self-reported information to their health records and are given control over their data by, for example, deciding who can access their health records [7,9,10,11]. The adoption of such systems, however, is a complex task. First, the implementation of electronic patient records has to be performed on the institutional level where all care providers have to adopt all technologies [1,12]. Secondly, the adoption of PHRs has to take place on the individual level. Individual attitudes are crucial on that level, as patients can reject opting-in to the PHR or demand to opt-out from the PHR [1]. Consequently, it is vital to understand how individuals will be willing to adopt such a system [13,14]. The patient’s choice in not adopting a PHR is crucial in the diffusion process [1] as patients’ resistance can result in “any national efforts unachievable” ([13], p. 360). Though the integration of an electronic health record serves potential benefits, it also constitutes risks for an individual’s privacy [15]. Privacy concerns remain the significant factor for patients in terms of withholding EHR adoption [13,14,16]. In particular, individual health information can easily be de-anonymized when combined [17]. Consequently, health and medical data’s highly sensitive nature results in many ethical issues when establishing a nationwide electronic health record [7].

The German healthcare system is characterized by fragmented care structures that hinder cross-sectoral care of patients and can lead to additional costs for the healthcare system, such as loss of information between practitioners, duplicated examinations, and uncoordinated treatment processes [18]. Germany’s electronic patient record project (*elektronische Patientenakte—ePA*) intends to overcome these barriers while increasing transparency and efficiency [19,20]. As of 1 January 2021, statutory health insurance companies in Germany have been obligated to offer electronic patient records to their insureds (§ 341 German Social Code, Book V). In an early study, Hoerbst et al. [21] gathered attitudes towards EHRs among Austrian and German citizens and found that citizens are generally interested in managing their health data and exchanging data between healthcare providers; however, data protection concerns were often mentioned. A Eurobarometer survey [22] showed that respondents generally like to have web-based access to their medical records, depending on the possibility of limiting access. Studies showed that privacy controls determined by the patient are a prerequisite for sharing health information [23,24]. A lack of granular controls negatively influences the willingness to share health information with other health professionals [25]. Consequently, worse healthcare outcomes can be expected [26]. Additionally, Caine and Hanania [27] discussed that patients want detailed privacy controls over their data in health records. That is why we aimed to understand different antecedents that add to an individual’s privacy concerns about ePA. For a more comprehensive view, this mixed-method study aimed to research individual’s attitudes towards the ePA by considering different types of motivations.

## 2. Theoretical Background and Prior Research

### 2.1. Endogenous Motivations in Driving Usage Intentions

In motivational psychology, it is the consensus that individuals’ motivations can be either intrinsically or extrinsically originated (i.e., [28]), which is also embodied in Davis et al.’s [29] motivational model, which represents the prevailing perspective on understanding user intentions [30]. In this model, “extrinsic motivation influences behavior due to the reinforcement value of outcomes, [while] intrinsic motivation refers to the performance of an activity for no apparent reinforcement other than the process of performing the activity per se” ([29], p. 1112). Consequently, “perceived usefulness is an example of extrinsic motivation, whereas enjoyment is an example of intrinsic motivation” ([29], p. 1112). Even though prevailing technology adoption models help to explain many antecedents to behavioral intentions, such as perceived usefulness and ease of use, traditional technology acceptance models fail to capture significance because of uncaptured “user-beliefs” [30,31]. That is why the extrinsic/intrinsic dichotomy that comes with the conceptualization of extrinsic motivation as perceived usefulness and intrinsic motivation as enjoyment may result in an incomplete understanding [31]. Therefore, a different approach for capturing user intentions was proposed by Malhotra et al. [31]. It utilizes *organismic integration theory* (OIT) [32], which is a sub-theory of *self-determination theory* (SDT) [33].

Historically, research has seen motivation as a concept that varies primarily in quantity (cf. [34]), the idea being that more motivated people “will aspire greater achievement and be more successful in their efforts than people with less motivation” ([35], pp. 221–222). In contrast, SDT argues that the different types of motivation are more vital than the level of motivation in predicting behavioral outcomes [33,36]. Thus, the “distinction between autonomous versus controlled is more important than the distinction between intrinsic and extrinsic” ([37], p. 471). The OIT regards motivation as the level of internalization and integration of the activity’s value [32]. Individuals that experience their behaviors as autonomously driven perceive volition, whereas individuals whose behaviors are linked to feelings of pressure recognize themselves as being controlled [35]. There is evidence that perceived volitional motivation has a more significant influence on the behavioral outcome than motivation through external influences [31,35,38,39].

**Figure 1:**
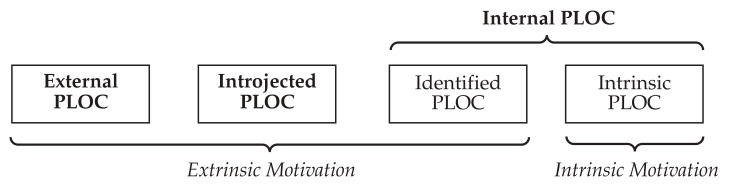
Different types of endogenous motivations and their relations to the PLOC notation (adapted from [31]).

The OIT specifies a taxonomy for the levels of perceived autonomy, referring to the *perceived locus of causality (PLOC)*. The PLOC describes the extent to which someone senses an action as being self-initiated [32]. Figure 1 shows the relations of different types of endogenous motivations to specific PLOC types. With an *internal PLOC*, individuals see themselves as the originators of their behavior, whereas with an *external PLOC*, people see themselves as being controlled by external forces [31]. For example, users may be motivated to learn how to use a new piece of technology out of self-interest or compliance with a supervisor. Internal PLOC further splits into *identified PLOC* and *intrinsic PLOC*. Feelings of volition are common to both types. Intrinsic PLOC refers to instinctive and spontaneous behavior [40] that results in actions being performed due to inherent enjoyment or fun [32]. Identified PLOC, however, refers to behavior based on individual values and meaningful goals that are performed freely and autonomously [40]. Because identified PLOC motivational behavior results from internalizing external regulations as essential values, this is a type of extrinsic motivation [31]. Both intrinsic and identified PLOC are often combined into a composite of autonomous motivation [31]. Both types imply an internal PLOC, but only identified PLOC can be directorially influenced motivation [31]. In *external PLOC*, individuals attribute the reasons for their actions to external authority or compliance [32]. A crucial characteristic of external PLOC is that perceived external influences and personal values are not conflicting [31]. *Introjected PLOC*, however, is defined by a misalignment of perceived external influence and personal values [31]. The conflict can result in affective feelings of guilt and shame or esteem-based pressure to act [32,40]. Introjected PLOC often leads to rejection of the “imposed” behavior [31]. Even though both external PLOC and introjected PLOC are linked to external influence, they result in different behavioral outcomes [32].

The PLOC framework [31] suggests that the different types of PLOC have cumulative effects on behavioral intentions. Understanding endogenous motivation can explain and predict individual differences in usage intentions across a population. The framework can also help explain different behavioral outcomes and why some users more widely accept some technologies than others. The PLOC framework has been applied in various research, such as in sustainable consumer behavior and educational and health-related lifestyle contexts [41,42]. Existing studies demonstrate that the PLOC framework needs to be contextualized. For a context-specific study, relevant contextual variables need to be used [43,44]. Consequently, we contextualized the PLOC model by conducting a qualitative study in the first phase of the mixed-methods design [45,46].

### 2.2. Privacy Theories and Research in the Health Context

The ubiquitous nature of information technology led to a “privacy is dead” shockwave [47]; however, many still consider health data sensitive and believe it should still be protected [15]. Even though the privacy literature is comprehensive, research in the health context is still ongoing. Only a few studies have examined human privacy in the health context (e.g., [1,2,15,48,49,50,51]). Existing literature demonstrates that protecting health data is increasingly vital to individuals. Privacy concerns result in privacy-protective behaviors, such as rejecting to adopt health solutions, including EHRs, [13,52,53,54], and holding back information from health professionals [2,55], which can negatively influence diagnoses.

The privacy concept has been discussed in various ways, but most literature emphasizes the matter of *control* [56,57,58,59,60]. For example, building on top of Clarke [61], Bélanger and Crossler [62] defined privacy as an individual’s desire for control over their personal information. Similarly, in the health context, Fox and Connollly [63] define privacy as an individual’s desire to be granted greater control over collecting and disseminating personal health information via health professionals and technology vendors. As the concept of human privacy remains challenging to measure, various other concepts are used as proximal measures. As such, *privacy concern* has been established as a central measure [62,64,65]. Privacy concern is the extent of the perception of a potential loss of privacy [66], i.e., the general tendency of people to worry about the loss of their informational privacy [67,68].

**Figure 2:**
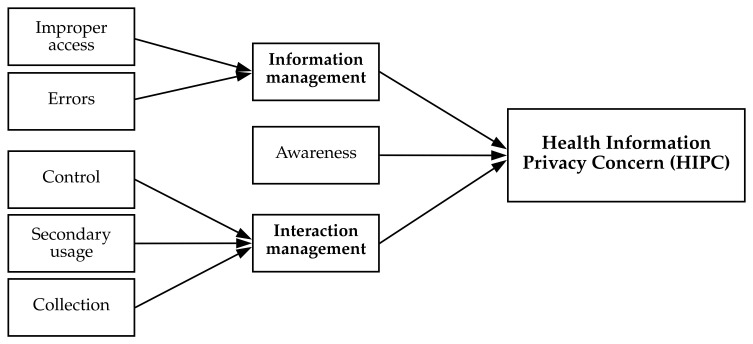
Formative third-order factor to measure the health information privacy concern (adapted from [15]).

To measure privacy concerns, Smith et al. [64] introduced a 4-dimensional “Concern for Information Privacy” (CFIP) scale, which queries individuals’ concerns regarding the collection, errors, unauthorized secondary use, and improper access of their information. The CFIP then has been used to measure health information privacy concerns in electronic medical reports and EHRs [1,13,16,52,54]. However, Kordzadeh et al. [48] suggested acknowledging additional factors of Internet Users’ Information Privacy Concerns (IUIPC) [68], namely, the sub-dimensions collection, control, and awareness. The CFIP and IUIPC were combined to a 6-dimensional “Internet Privacy Concerns” (IPC) scale by Hong and Thong [69]. Fox and Connolly [63] then rephrased the IPC measure to create the Health Information Privacy Concern scale. Hong and Thong [69] formed the IPC as a third-order construct, and as a consequence, the HIPC has three dimensions as well. Concerns about collection, secondary usage, and control form a second-order interaction management factor, and errors and improper access constitute the second-order factor information management. Both second-order factors, plus awareness, build the third-order factor (H)IPC. The HIPC is shown in Figure 2. While past studies on healthcare adoption often measured privacy concern with one dimension (cf. [14,48,70]), the complex nature of m-health technology requires a more sophisticated approach to measuring human privacy. The HIPC is multidimensional, as depicted in Figure 2, and is preferably measured as such [63,69].

Prior research has mainly utilized the antecedents→privacy concerns→outcomes (APCO) macro model that posits that a number of *antecedents*, usually individual traits or contextual factors, form an individual’s privacy concerns, which, in turn, cause behavioral outcomes [65]. However, the majority of studies focus on the outcomes rather than on the antecedents [65]. A major contribution to the research of antecedents on the HIPC has been made by Fox and James [15]. This work will further examine antecedents that are subject to impact the HIPCs while simultaneously validating the factors found by Fox and James [15].

### 2.3. Risk and Trust Beliefs in Privacy Research

Besides privacy concerns, *risk* and *trust* play significant roles in privacy research. *Trust beliefs* become crucial when dealing with uncertainty [71], where trust is the “belief that the trusted party will fulfill its commitments [72,73] despite the trusting party’s dependence and vulnerability [74,75]” ([76], p. 54). Research shows that greater trust in the vendor’s *competence*, *benevolence*, and *integrity* results in lower privacy concerns [77,78,79]. Research in the health context supports the influence of trust on privacy concerns. For instance, Bansal et al. [14] found that trust influences the customer’s willingness to interact with health-related websites. Dinev et al. [1] also found that trust in EHR system vendors reduces privacy concerns. On the other hand, *risk* is considered an antithesis to trust and can be described as one’s expectation that information disclosure will have a negative outcome [56]. Studies have shown that risk perceptions increase privacy concerns for health websites [50] and reduce usage intentions for health-promoting wearables [80].

For electronic health records, users expect a heightened probability of privacy breaches and data misuse [13]. Additionally, “the highly sensitive nature of personal medical data adds even more to the uneasiness individuals feel about the violations and misuse” ([1], p. 29). Those concerns are general and are not necessarily linked to specific systems or practices [1]. Consequently, trust is crucial in overcoming risk perceptions concerning electronic health records [1,14]. McKnight et al. [79] distinguished between institution-based trust and disposition to trust in information systems research. For EHRs, an individual can trust a health professional but may not necessarily trust EHR systems; alternatively, an individual may value an EHR but not the institutions or care providers using it [1].

### 2.4. IT Identity in Predicting IT Adoption Intentions

Self-categorization and social comparison shape an individual’s identity [81]. Self-categorization supports individuals in putting their social environment into order and understanding and recognizing their peers [82]. The self-identity develops over time as people observe and categorize themselves relative to others based on their goals, perceptions of how others respond to them, and their self-evaluations [83]. One type of self-categorization is to see IT as being integral to the sense of self. Carter and Grover ([84], p. 938) defined IT identity as “the extent to which a person views use of IT as integral to his or her sense of self”. The concept assumes that an individual’s IT usage is motivated by positive self-identification with IT use [85]. People who highly self-identify with IT employ IT usage more often than those who do not identify with the technology [84].

Carter [86] defined three dimensions of IT identity that serve an individual’s self-perception about IT: dependency, emotional energy, and relatedness. *Dependency* is specified as “the degree of reliance a person feels on a particular IT or class of ITs as a source of personal well-being” ([86], p. 115). IT is so ubiquitous that businesses and humans depend on it, so it constitutes one component of individuals’ identities. People express the perception of a need for devices. *Emotional energy* is defined as “an individual’s enduring feelings of emotional attachment and enthusiasm in relation to an IT or class of ITs” ([86], p. 115). For example, continuous interaction with an IT device could result in confidence, energy, and enthusiasm. Conversely, lack of these emotions can cause negative feelings, such as boredom [87]. Finally, *relatedness* refers to “a blurring of boundaries between notions of the self and an IT experienced as feelings of connectedness with an IT or class of ITs” ([86], p. 114). When individuals incorporate their devices’ characteristics within their self-identities, they feel intimately connected to these IT devices.

In the digital health context, it was proposed that IT identity influences emotions relating to IT, affecting patients’ decisions on whether or not to adopt healthcare devices or applications [88]. Additionally, the literature indicates that the IT identity theory is a relevant factor in explaining patients’ interaction with m-health applications [89]. Accordingly, this work adopts IT identity and its dimension to predict users’ adoption intention of the ePA application.

## 3. Prototype

**Figure 3:**
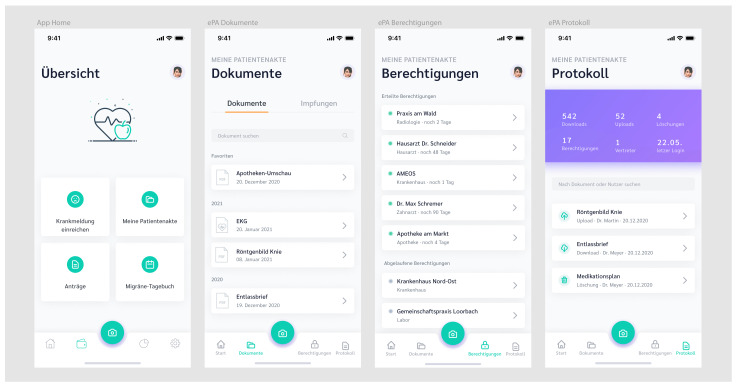
The prototype of the ePA application used in our research to inform the participants.

At the time of research, the ePA had not been launched, and as we write now, the ePA has not had high diffusion due to missing technical infrastructure [90]. To overcome this limitation, we reviewed the ePA and provided a prototypical ePA mobile application. Based on the findings in the literature (e.g., [91]), we developed a prototype to be used for further research and present it in Figure 3. The prototype was used to inform the participants of the following studies. Creating a distinct prototype will also help to create a common understanding of the ePA, which should be helpful, given the breadth of available ePA applications. For prototyping, we utilized Figma [92]—a “mid-fidelity” prototyping tool for creating interfaces that can be immediately tested to get practical impressions of the applications [93].

As each health insurer will provide its own version of an ePA application, the applications will be branded. Health insurance companies and technology vendors likely offer ePAs as parts of more comprehensive digital health applications. All electronic documents in the patient file are listed chronologically by name, and by publication date in the document view (second screen). With the ePA being patient-administered, users can upload any documents, even newspaper articles. Another view (third screen) visualizes the permissions screen where all given permissions are listed. The list gives an overview of what health providers were given access, in the past or continuing. The last screen holds a record of every action performed on the patient file. For instance, the list gives an overview of what files have been uploaded, downloaded, deleted, and by whom.

## 4. The Mixed-Methods Design

We applied a two-stage sequential mixed-methods design to research the intentions, attitudes, and privacy concerns towards ePAs. Mixed-methods research combines “elements of quantitative and qualitative research approaches [*…*] for the broad purposes of breadth and depth of understanding and corroboration” ([94], p. 123). A mixed-methods design approach is compelling in the ever-changing IT context, where researchers encounter problems with the explanatory power of existing theories and findings [30]. Mixed-methods research offers three main advantages: it allows one to address confirmatory and explanatory research questions simultaneously, provides more robust inferences than a single method, and can produce a more comprehensive range of divergent and complementary views [46].

**Figure 4:**
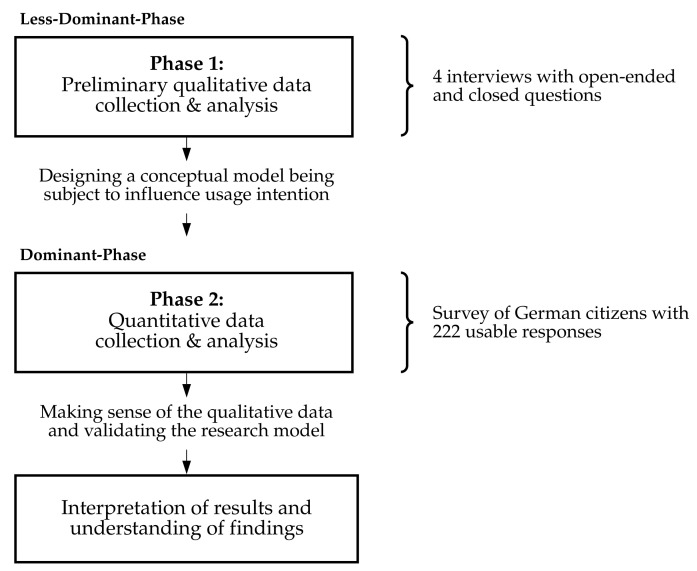
The mixed-methods multistrand exploratory-explanatory research with the dominant-less dominant design.

The overall study made use of Venkatesh et al.’s [46] design guidelines. At the beginning of the process, we defined three research questions (one qualitative, one quantitative, and one mixed-methods; see Appendix A). The purpose of the mixed-methods is “developmental,” where the findings from the first strand qualitative method are used to inform the second strand quantitative method [46]. This study followed multiple paradigms from an epistemological perspective, with the first strand being interpretive and the second strand being deductive [46]. The methodology is “mixed-methods multistrand” ([46], p. 443) with a “sequential exploratory design” [95], which is characterized by the qualitative phase followed by its quantitative phase ([46], p. 445). The research design is sequential exploratory-explanatory, as it combines exploratory and explanatory approaches [96]. The study falls into the category of a “dominant-less dominant design,” with the quantitative strand being dominant in the overall design ([97], p. 44). Appendix A proves the design choices made. Figure 4 visualizes the dominant–less dominant design of our mixed-methods study.

## 5. Phase 1 Qualitative Study

The phase 1 qualitative study aimed to answer the research question: “What are the salient factors determining an individual’s intentions toward using the ePA?” For answering this question, we conducted semi-structured interviews with four individuals.

### 5.1. Research Methodology

Before the interviews, we created a semi-structured interview guideline. Semi-structured interviews encourage communication, thereby encouraging respondents to reveal underlying concepts [98]. The conversational form allows follow-up questions and prompts based on the answers [98]. This approach is particularly appropriate for generating new theories rather than confirming the established theory. The four interviewees (Appendix C) were identified through purposive sampling, i.e., non-probabilistic sampling where subjects were selected intentionally [99]. The interviews took about 30 min each and were conducted in German with a combination of open-ended and closed questions (Appendix B). All interviews were conducted remotely, recorded, and transcribed. Ethical considerations included measures such as only using encrypted communication channels, using pseudonyms in the transcripts, and not asking for health-related circumstances, such as chronic diseases. Beginning with some general questions, we presented the prototype of the ePA application from Figure 3. The prototype was explained in detail but took no longer than five minutes. Subsequently, the interviewees were asked about general attitudes toward this application and were asked to articulate how these attitudes were constituted. Subsequently, the respondents were asked about their health information privacy concerns (cf. [15]). Other questions cover the interviewee’s usage intentions, perceived benefits, and perceptions of risk.

We used an inductive approach [100] to make sense of the interviews rather than quantifying the data. We started by generating a list of “start list” of codes ([101], p. 58) resulting from the literature review. Then, with a “constant comparative” ([102], p. 105) analysis, we intended to identify the initial concepts and to link them to resulting sets of broader categories [103]. In grounded theory methodology, this procedure is equivalent to the “open coding” phase ([104], p. 12), where “conceptually similar events/actions/interactions are grouped to form categories and subcategories”. We used the software Atlas.ti to apply codes to the transcripts. Through constant comparison, “abstract categories” of labels were assigned to similar concepts ([101], p. 58).

### 5.2. Findings

The coding of the transcripts revealed different types of motivation-related variables. Identified PLOC was emergent, i.e., interest in accessing health data or more efficient treatments. Additionally, coding revealed that advice from health professionals supports ePA usage intentions, indicating that external PLOC drives adoption intentions. Introjected PLOC can result in rejecting the ePA, as respondents discussed that a negative medical history could result in feelings of shame due to conflicting external expectations in internal values. Among these motivation-related variables, respondents mentioned that a person’s IT experience and age could influence the usage intentions. In the interviews, we found indicators for different privacy concerns, i.e., concern for collection, secondary usage, improper access and errors, and a general desire for privacy and control over their data. Especially regarding the desire for control, many respondents underlined the importance of granular access rights. The trade-off between privacy risk and trust was repeatedly mentioned. Respondents mentioned that the trust regarding their physicians positively influences their intentions and perceived risk, especially regarding the general data collection practices on technical devices, has a negative impact. Among these variables, some interviewees mentioned that usability might play a role in using or discontinuing use of the application. The topics and broader concepts that were emergent through the interviewees are provided in Appendix D. Additionally, Appendix E displays clarifying quotes per the interviewees.

## 6. Research Model

The research model used (1) the PLOC framework [31] as the underlying theory to capture individual motivations, (2) the HIPC construct with contextualized privacy antecedents, and (3) heuristically evaluated context-based constructs to develop and justify the hypotheses. Table 1 shows the constructs and their definitions. We used *intention* as the dependent variable because an intention is the most “proximal” influence on behavior ([105], p. 76). An intention is what one plans on doing.

Internal PLOC is identified by the intrinsic and the identified PLOC that both. Feelings of volition characterize both states. Intrinsic PLOC refers to spontaneous behavior and performance for inherent fun, and identified PLOC refers to behavior based on personal values, goals, and outcomes [32]. For the ePA, users may adopt it if they can control it (intrinsic drivers) or be guided by internalized values such as health awareness. For example, one interviewee (I1) said that they likes to see “which current diagnoses I will have or which doctor’s letters and documents come together that exist about me”. Hence,

**Hypothesis** **1.** *Internal PLOC positively influences one’s intentions toward adopting ePA applications.*

External PLOC is perceived when one’s actions are attributed to external authority [32]. There must be no conflict between the perceived external influences and an individual’s internal values. The resulting behavior is usually done to comply with external demands. In the case of the ePA, such external demands could arise from recommendations by physicians or the health insurance. Hence,

**Hypothesis** **2.** *External PLOC positively influences one’s intentions toward adopting ePA applications.*

Introjected PLOC refers to feelings of shame and guilt that may emerge from other parties prompting them to act in a particular way (e.g., [33]). The user feels tension and confusion as introjected PLOC derives from misalignment between a user’s beliefs about behavior and their self-perceived autonomy [32]. If users experience that either their health insurance or the government is exerting pressure to use the ePA but judge themselves to be autonomous, the resulting uncertainty is likely to influence the usage intentions of ePA applications negatively. Hence,

**Hypothesis** **3.** *Introjected PLOC negatively influences one’s intentions toward adopting ePA applications.*

As to Carter and Grover [84], there are three behavioral consequences of IT identity: feature use behavior, enhanced use, and resistance behavior. Consequently, mobile technology identity can lead to both resistance and adoption [84]. Higher mobile technology identity can lead to higher motivation to adopt mobile applications, since people are dependent on and enthusiastic about their phones. This enthusiasm concerning the mobile device can increase the individual’s motivation to adopt m-health applications [107]. Additionally, feelings of IT dependence or relatedness can motivate people because they can link these feelings to dimensions of their identity [84]. Hence,

**Hypothesis** **4.** *Mobile technology identity positively influences one’s intentions toward adopting ePA applications.*

The interviews indicate that age has a role in the adoption of ePA applications. One interviewee, being aged 50+, expressed that they is very unfamiliar with technology and media, resulting in being conservative (I4). Another interviewee noted that older people might have problems with handling such applications and would not use the ePA. In literature, demographics, such as age, are commonly associated with privacy concerns. In Laric et al. [70], older participants expressed deeper privacy concerns regarding healthcare services. In contrast, Kordzadeh et al. [48] found younger users to have more significant privacy concerns attributed to their privacy literacy. In Vodicka et al. [108], people under 55 expressed more severe privacy concerns of the physician’s notes from their treatments. Additionally, King et al. [109] found that age correlates with concern about health information privacy. The majority of studies have revealed that privacy concerns increase with age, which is in line with the findings of the phase 1 study. Hence,

**Hypothesis** **5.** *Age positively influences the HIPC.*

The health status was an emergent theme in our interviews. While one interviewee stated that they would use the application uncoupled from their health status, other interviewees expressed concerns. For example, one interviewee stated that people with certain chronic diseases retain from using the ePA:

People with serious chronic illnesses, psychological problems, and those who fall under social taboos will hardly use the app.

(I3)

People with severe medical conditions require frequent treatments [110]. Thus, those people generate the most personal health information and are likely to express higher privacy concerns [15]. In Flynn et al. [111], people who feared mental illness’s stigma were less likely to opt into an electronic psychiatric record. Other studies support that health status influences information sensitivity and privacy concerns [14,112]. Based on the literature and phase 1 study findings, we posit that severe health conditions have impacts privacy concerns. Hence,

**Hypothesis** **6.** *A severe health condition positively influences the HIPC.*

Anderson and Agarwal [2] claimed that perceived information sensitivity affects privacy concerns and intentions to provide personal health information. In Dinev et al. [56], information sensitivity was associated with perceived risk. Caine and Hanania [27] found that the decision to share data in an EHR with some particular parties was based on the perceived sensitivity of personal health information. Additionally, Bansal and Davenport [14] found a positive correlation between health information sensitivity and privacy concerns. Further, the “highly sensitive nature of personal medical data” increases one’s concerns of violations and data misuse ([1], p. 29). One respondent demonstrated this:

If it says in your documents, you have some sexually transmitted disease or something, you may not want everyone to access it because it’s something that’s only your business.

(I2)

With support from the literature and the qualitative findings, we posit that perceived sensitivity of health information impacts privacy concerns. Hence,

**Hypothesis** **7.** *Perceived information sensitivity positively influences the HIPC.*

Trust and risk often are linked to privacy concerns [1,66,113]. Even though the APCO model positions risk as an outcome of privacy concerns, Smith et al. [65] recognized that prior studies support the influence of privacy risk on privacy concerns. Studies have shown that perceived privacy risk positively correlates with different websites, including healthcare ones [50]. In the case of the ePA, both health professionals and health insurance companies handle personal health information. Hence,

**Hypothesis** **8a.** *Perceived risk associated with health professionals positively influences the HIPC.*

**Hypothesis** **8b.** *Perceived risk associated with health institutions positively influences the HIPC.*

Additionally, trust has been shown to both an outcome and an antecedent of privacy concerns [65]. Prior studies found that trust in physicians and EHRs lowers privacy concerns [1,114]. One respondent in the qualitative study expressed that trust in their health insurer was a factor in using the ePA:

I would trust the health insurance companies. That plays an essential role for me.

(I1)

Hence,

**Hypothesis** **9a.** *Trust in health professionals negatively influences the HIPC.*

**Hypothesis** **9b.** *Trust in health institutions negatively influences the HIPC.*

Past studies show evidence that privacy concerns influence usage adoptions for health applications, including EHRs [15,53,115]. We consequently posit that with an increased HIPC, individuals will be less likely intend to opt-in to the ePA. Hence,

**Hypothesis** **10.** *The HIPC negatively influences intentions to adopt ePA applications.*

**Figure 5:**
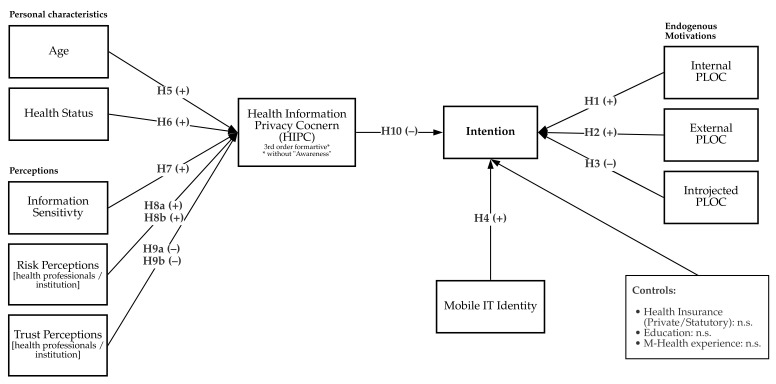
The research model with the different hypotheses.

Besides these variables mentioned, the questionnaire of the phase 2 study also covered traditional control variables such as education, employment, as the literature advocates that these elements affect behavioral intentions [30,116]. Thus, those factors were added to the questionnaire to enrich understanding of the ePA applications’ usage intentions. Finally, we present the research model conceptualized from the hypotheses in Figure 5.

## 7. Phase 2 Quantitative Study

The second phase of the mixed-methods study aimed to answer the question, “Does the research model explain usage intentions of the ePA?” Therefore, we conducted a survey of potential German adopters to test the research model.

### 7.1. Research Methodology

To gather empirical data from potential adopters, we conducted an online survey with a traditional questionnaire design consisting of a closed-questions design. The questionnaire was published on SoSci Survey. The advantages of an online survey are a potentially broader target audience, straightforward distribution and analysis, and the collection of additional measures, such as the time needed to complete the survey. The survey was distributed via e-mail to a list of acquaintances and a market research panel simultaneously to gather responses from participants with diverse socio-demographic backgrounds. To be included to the study, participants needed to be at least 18 and have a permanent residency in Germany. At the beginning of the survey, we presented the screenshot of our prototypical ePA application (Figure 3) with a brief explanation of available features to establish a common understanding of the ePA.

The research model was analyzed using partial least squares structural equation modeling (PLS-SEM). The calculations were made in SmartPLS version 3.3.3 [117]. PLS regression is often used in information systems research to understand behavioral phenomena. We applied current recommendations and validation tools to analyze our model [118].

### 7.2. Measures and Pilot Testing

For measuring intention to use the ePA, we used a two-item scale based on the literature [30,119]. The endogenous motivation was measured by scales based on Ryan and Connell [32] that were extended to capture ePA adoption. Those measures were greatly influenced by the findings of the phase 1 study. Items for external PLOC measured self-perceived reasons for usage intentions resulting from their health insurance or physician recommendations. The internal PLOC scale measured reasons for using an ePA characterized by self-determined choice and volition. For measuring introjected PLOC, we used items dealing with conflicts between personal values and social norms. We added two-item scales to measure both dependence and emotional energy as characteristics of IT identity [84,86]. For measuring an individual’s Health Information Privacy Concern in the ePA, we added a three-item scale for each dimension—secondary usage, control, errors, and improper access from Fox and James [15,69]. We, however, omitted the *awareness* construct of the HIPC scale since we did not find evidence for this attribute in the interviews. In addition, the questioning focused on the individuals’ perceptions of their concerns rather than their expectations, as proposed by Hong and Thong [69]. Capturing the individual’s health status involved a three-item scale based on Bansal et al. [14]. We utilized a two-item scale for each category to capture risk perception concerning health professionals and insurance providers and capture trust towards health professionals and insurance providers [15,53,69]. For measuring personal health information sensitivity, we utilized a 5-point-Likert-scale for different categories of health data to rate the perceived sensitivity, based on Laric et al. [70]. Finally, we added items for measuring the demographic characteristics age (offering four categories), employment (four categories), formal educational level (four categories), and prior m-health experience (yes/no). At the end of the survey, we added items that gathered self-reporting seriousness checks to improve data quality [120]. All scales are presented in detail in Appendix F.

The survey used validated construct scales from the literature where applicable. Several best practices were applied to avoid common-method bias [121,122,123]: The questionnaire was designed to maximize user engagement and minimize task difficulty. A “good cover story” ([123], p. 562) on the opening page of the survey aimed to engage respondents’ accuracy and motivation. The introductory text was both descriptive and motivating by emphasizing the respondent’s desire for self-expression [123]. We applied clear and concise language and avoided ambiguous or unfamiliar terms. We separated parts in the questionnaire and repeatedly displayed the image of the prototype in the hope of diminishing “effects of involuntary memory-based and perceptual biases” ([123], p. 563). The questionnaire was pilot tested to validate the instrument. We conducted two on-site and two remote pilot tests. The user tests provided feedback that resulted in the rewording of items and clarified descriptions. Participants reported difficulties with one item from the introjected PLOC scale that we decided to drop from the questionnaire. The questionnaire was then reviewed until judged satisfactory.

### 7.3. Sample

The sample was intended to be “probabilistic” [46]. The heterogeneity of the sample could be verified by the descriptive analysis of the survey data. The external validity of the sample was reasonably ascertained by assuring that the sample represented the whole German population by comparing the sample with data of German citizens (see Appendix G). The online survey was opened 480 times. A total of 289 participants commenced the survey, which makes a response rate of 60%, though the click-rate of the survey is a vague metric. Among those participants, 250 respondents finished the last page of the survey. Then, incomplete responses (*n* = 2) were removed, which resulted in a completion rate of 86%. For data cleaning, we followed the practice that all cases should be retained unless evidence suggests a case is aberrant [124]. Leiner ([125], p. 242) proposed a “relative speed index” to eliminate potentially meaningless cases by completion time. We chose a speed index of 2.00 and removed *n* = 17 responses with completion times two times faster than the median completion time. We also removed cases from respondents who did not give their consent or self-reported their answers as meaningless. After data cleaning, 222 responses were used for further analysis. Participants’ demographic characteristics are shown in Appendix G. Basic descriptive characteristics (mean, standard derivation) are presented in Appendix F.

### 7.4. Preliminary Analysis Validation

To ascertain the quality of the quantitative results, we evaluated a range of reliability measures to test the convergent and discriminant validity of the scales [121]. We began with assessing the convergent validity by evaluating the multi-item construct quality (see Appendix H). The Cronbach’s alpha revealed undesirable internal consistency (α<0.600) for both the introjected PLOC and HIPC-control scale. A low alpha indicates poor inter-relatedness between items or heterogeneous constructs [126]. For both scales, we improved internal consistency by dropping one item from the construct. We then further assessed construct reliability by conducting the composite reliability and AVE scores. The composite reliability should exceed 0.700 and be larger than the AVE [124], which was the case for all constructs. We further obtained the outer loadings and t-statistics for all items across each construct. Loadings above 0.700 are often recommended, but lower values can be sufficient [124]. As Appendix I reports, all items had outer loadings above 0.700 and were significant at the p<0.05 level.

We also calculated the tolerance levels and variance inflation factors (VIF) to check for multi-collinearity. The threshold of 10 [127] was passed for two of the HIPC-access items. The tolerance levels were all greater than 0.10, but for the before-mentioned items, thereby indicating that multicollinearity generally is no issue. As these two items are used to form a third-order factor to measure HIPC, we did not consider the VIF problematic. Thus, all items were retained for further analysis.

To examine the discriminant validity, we conducted a Fornell–Larcker test (see Appendix J). A latent construct should better explain the variance of its indicator than the variances of other latent constructs [121,128]. The average variance extracted (AVE) from each of the latent constructs should be higher than the highest squared correlation with any other latent variable. Our test ensured that the square root of the AVE exceeded all correlations with other latent constructs, and discriminant validity was given.

### 7.5. Model Results

**Figure 6:**
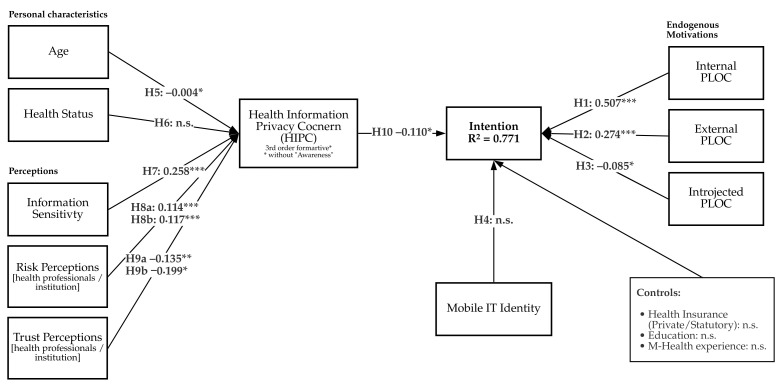
Summary of Full Model Results. ${}^{*}p<0.05;$${}^{**}p<0.01;$${}^{***}p<0.001$.

The structural model results are summarized in Table 2 and Figure 6.

## 8. Discussion

The mixed-methods design aimed to discover individual’s intentions toward using the ePA mobile applications. The qualitative study uncovered a range of factors influencing usage intentions to formulate 12 hypotheses. The results of the quantitative study show overall support for most hypotheses. We implemented qualitative analysis, followed by the quantitative analysis (see Table 3) [46]. The results show consistency but also reveal some incompatible findings. Overall, we found the same row of parameters were significant in both qualitative and quantitative studies. Even though the questionnaire was developed from the findings of the qualitative study, we found some significant differences in the findings of the studies: Besides an individual’s health status emerging as a critical factor for ePA adoption in the qualitative study, health status was not significant in the second study. Similarly, one’s positive self-identification with mobile devices (“mobile IT identity”) was not significant in the quantitative study. A limitation of our study is that we did not replicate the divergent results with a new dataset [46,129]. However, we offer a theoretical explanation to remedy the inconsistent findings.

Overall, our meta-inferences are congruent with our research model. We successfully added value beyond the individual studies with the integration of the qualitative and quantitative research strands. Considering that the phase 1 and phase 2 study data were from different sets of respondents and different data-collection approaches, the similarity implies that we utilized solid theoretical models as our research foundation. The mixed-methods helped us determine and understand factors that influence ePA usage intentions. With the qualitative study, we were able to determine a set of aspects and their relevance. In contrast, the quantitative study empirically examined the research model that resulted from the qualitative study to determine what factors influence ePA usage intentions. Table 3 summarizes our meta-inferences.

In particular, the results highlight the predictive power of motivation. Some respondents from the phase 1 study expressed intrinsic PLOC, i.e., “joy” in accessing their data and using the ePA. Other respondents expressed indicators that relate to the perceived usefulness, thereby relating to identified PLOC. For example, one respondent identified that a digital health record helps them to keep track of their data, even when consulting different physicians:

I have moved several times in my life now, even long distances. In the end, I always had to have everything handed over to me in physical form by the family doctor I was seeing.

(I3)

Both intrinsic and identified PLOC were crucial factors for predicting ePA usage intentions among the studies. Some respondents indicated that they considered adopting the ePA when advised to, indicating the motivational power of external PLOC. Those findings were consistent among both strands of our mixed-methods study. However, internal PLOC was a stronger predictor than external PLOC. These findings are consistent with the literature about external rewards [39,130]. We found strong indicators for introjected PLOC, hindering ePA adoption in the qualitative study. The respondents repeatedly expressed uneasiness resulting from a misalignment of perceived social influences and personal values:

I think if you are seriously ill and you carry this application around with you all the time, it’s like carrying your X-rays around with you all the time. I don’t like the idea.

(I2)

People with serious chronic illnesses, psychological problems, and those who fall under social taboos will hardly use the app.

(I3)

The quantitative study supported the negative impact of introjected PLOC. In particular, political pressure and shame were two factors that hindered ePA adoption from the quantitative study.

Contrary to our expectations, the meta-analysis for the “mobile IT identity” and “health status” variables indicate the lack of influence of these factors on ePA adoption or the HIPC. We now attempt to explain these meta-analysis: (1) The low impact of one’s mobile IT identity can be explained by the not-so-technical nature of a health record: Even though the ePA is distributed as a mobile application, such an application does not require a self-identity that is usually attributed to “mobile IT identity”. We assume that, in contrast, ePA applications being heavily gamified might demand positive perceptions towards IT in a pronounced manner. (2) An individual’s health status did not have a significant influence on the HIPC. The share of subjects with self-reported severe health status was generally low in our sample; thus, our quantitative study failed to see an effect on this variable. We argued that people with severe health conditions would express higher privacy concerns; however, none of the interviewees from Study 1 reported severe chronic diseases themselves, but thought that there might be concerns from people with such conditions. On the other hand, populations with multiple chronic conditions may have more motivation to use the ePA to facilitate patient–doctor communication and control privacy settings themselves. Whether a severe health condition has a positive impact on privacy concerns, or a positive impact on the usage intentions, or even both, is not supported by the meta-analysis.

The quantitative study showed evidence that age has an impact on the HIPC. However, contrary to our assumptions, higher age led to lower HIPC. Our literature review showed conflicting findings for the impact of age on an individual’s privacy concern in the health context. One supported explanation is that younger people might have higher privacy concerns attributed to their privacy literacy, which is also supported by the literature [48].

The findings towards perceived risk and trust concerning health professionals and one’s health insurance were congruent among both strands of research. This is a strong indicator of risk and trust being linked to privacy concerns. Our findings are consistent with the literature [1,66,114,131]. For instance, a satisfying experience with one’s health insurance can lead to less resistance when adopting an ePA that is distributed by their health insurance, as stated by one respondent:

I have personally been very, very satisfied with my health insurance company over the years. I am sure that it works well, and I can download the application with confidence. In contrast, for third-party providers, I would have to deal with who is behind the app.

(I3)

Even though the APCO model positions risk as an outcome of the privacy concern, we demonstrated that privacy risk influences privacy concern. This impact was also theorized by Smith et al. [65].

Our findings from both research strands show that the attributed information sensitivity of health data adds to the HIPC towards the ePA. Individuals that perceived their health information as being more sensitive were less likely to adopt the ePA. This finding is consistent with the literature [2,14,27,56]. Overall, the information sensitivity and general demand for privacy differed among the respondents in the qualitative study, which was also reinforced by the quantitative findings. We discussed that the perceived privacy risk and privacy calculus are less profound where electronic patient records are relatively new. Individuals tend to weigh the benefits of the ePA more heavily than the concerns of privacy. However, those societal values may change over time during the diffusion process of the ePA. Additionally, secondary usage of one’s health data can result in uneasiness, for instance, when health data are used for data mining purposes, or when the data impact the services delivered by the health insurance.

In the qualitative study, the control that one could exercise over their health data was an essential factor in ePA usage intentions:

I would like to decide what the doctor can get from me and what insight he can get from me.

(I4)

Additionally, existing literature demonstrated that “patients want granular privacy control over health information in electronic medical records” [27].

The concept of perceived ownership of data in the ePA was also present in our interviews. For instance, one respondent mentioned that they did not feel up to exerting control over their data:

Do I wish I had control over it myself when my family doctor has the data? I would like to have confidence that the control will be realized by someone else.

(I2)

Tang et al. ([132], p. 125) noted that, with the patient having data sovereignty, “different mindsets and levels of trust” will become mandatory. Fox and James [15] researched the HIPC and found that interviewees have differing perceptions of data ownership in the context of EHRs. Perceptions ranged from beliefs that the patient is the owner, to dual ownership, to the perception that the physician owns the data. Fox and James [15] also found that interviewees seeing themselves as single data owners expressed a strong desire for privacy and were highly concerned about unauthorized secondary use, improper access, and control.

Our research model could explain 77% ($R^{2}=0.771$) of the variance in our sample, which is a satisfactory fit of our theoretical model. Thus, our study demonstrates that we used solid underlying theory, i.e., the PLOC framework, to understand the intentions toward adopting the ePA.

## 9. Limitations and Future Research

Our study has some limitations, which should create opportunities for future research. First, we interviewed four individuals in the first study, making it unlikely to reach theoretical saturation. Since study 1 was less dominant in the overall study design, this limitation was maintainable. Further, we did not ask health-status-related questions out of ethical considerations, which may have resulted in an incomplete picture of the impacts of health-related factors on ePA usage intentions. Next, we showed and described the prototype from Figure 3 to the participants in a detailed manner. However, the prototype was static, and the remote setting has downsides regarding user comprehension. Further studies should prepare a high-fidelity prototype and consider the impacts of participants’ digital skills and literacy levels.

Second, our sample in study 2 contained 222 usable responses from German citizens. Even though we ascertained external validity employing the demographics (see Appendix G), our sample had an imbalance in the age distribution. Further, we noticed that the share of respondents that reported severe health conditions was generally low. Additionally, an online survey requires a certain level of IT literacy. This is particularly important because a severe health status, IT literacy, and old age may co-occur. Similarly, we measured health status with a two-item scale (see Appendix F) that lacked understanding of the population’s actual health conditions. The scale used in the questionnaire mixes chronic and acute diseases from participants and does not capture multimorbidity, nor polypharmacy. Further studies should improve the measures to capture health status.

Third, even though we discovered several antecedents that impact privacy concerns, we did not capture each antecedent. This limitation has been shared by other studies examining the antecedents of the HIPC [15]. We thus encourage exploring privacy antecedents more comprehensively. Lastly, an individual’s perceptions evolve due to changing societal values or recent events. In addition, perceptions change over the time when a new technology is in the process of diffusion. Currently, Germany’s ePA is in an early testing stage. Even though some issues were already discussed in public, the concept of the ePA is not widespread yet. Thus, future research could apply a longitudinal study to get further insights into users’ adoption intentions.

## 10. Conclusions

The adoption of the ePA is a complex task. With the launch of new technology, such as the ePA, its adoption faces significant challenges. With a mixed-methods design and by developing a contextual model, we gathered evidence that different types of motivation, the HIPC, and privacy antecedents affect usage intentions regarding ePA. Most importantly, a profound understanding of the different types of motivation is critical to understanding individual usage intentions, since motivational variables were shown to explain the majority of the variance in our sample.

The findings showed the integral positive effect of internal PLOC. Individuals who feel volitional about using an electronic health record are more likely to adopt it. Consequently, policymakers must understand what types of motivation are critical predictors in ePA adoption and use. The findings demonstrate that policymakers have to provide both internal and external incentives. We believe that the results of this work contribute to the growing body of research on technology adoption in the field of the ePA in a German context.

## Figures and Tables

**Table 1:** Constructs and their definitions.

Construct	Definition
Intention toadopt the ePA [106]	The subjective probability that a person will perform the behavior of adopting ePA.
Internal PLOC [31]	Motivation stemming from feelings of volition where consumers perceive autonomy over their behavior.
External PLOC [31]	Motivation stemming from perceived reasons that are attributed to external authority or compliance. No conflict between perceived external influences and personal values exists.
Introjected PLOC [31]	Motivation due to a misalignment of perceived social influences and personal values often relates to guilt and shame. The conflict between esteemed pressures and the desire for being autonomous often results in rejection of the “imposed” behavior.
Mobile IT Identity [84]	The extent to which a person views IT or their mobile phone as integral to their sense of self.
Health Information PrivacyConcern (HIPC) [15,69]	An individual’s perception of their concern for how health entities handle personal data.
Health informationsensitivity [70]	The perceived sensitivity of an individual’s different health information.
Risk perceptions [15,56]	The perception that information disclosure towards health professionals or health insurance providers will have a negative outcome.
Trust perceptions [15,76]	The belief that health professionals or health insurance providers will fulfill their commitments.
Age	The age of the insurant.
Health Status	An individual’s reports of severe health conditions.
Education	The level of formal education of the insurant.
Employment	Employment status.
M-Health experience	An individual’s experience with health-related technologies and applications, i.e., wearables and health-supporting applications.

**Table 2:** Results for test hypotheses and control variables.

	Path Coef.	T Statistics	*p*-Values
H1: IPLOC → Intention	0.507	7.072	0.000
H2: EPLOC → Intention	0.274	3.340	0.001
H3: IJPLOC → Intention	−0.085	2.318	0.021
H4: IT Identity → Intention	0.011	0.293	0.770
H5: Age → HIPC	−0.004	2.556	0.011
H6: HealthStatus → HIPC	0.011	0.873	0.383
H7: InfoSensitivity → HIPC	0.258	5.299	0.000
H8a: RiskHP → HIPC	0.114	8.757	0.000
H8b: RiskIn → HIPC	0.117	8.983	0.000
H9a: TrustHP → HIPC	−0.135	2.870	0.004
H9b: TrustIn → HIPC	−0.199	2.330	0.020
H10: HIPC → Intention	−0.110	2.096	0.036
*Controls:*			
Education → Intention	−0.023	0.702	0.483
Prior m-health experience → Intention	0.009	0.230	0.818
Health Insurance → Intention	−0.045	1.222	0.222

**Table 3:** Development of qualitative inferences, quantitative inferences, and meta-inferences (adapted from [46]).

Context and Category of Constructs	Specific Construct	Qualitative Interference	Quantitative Interference	Meta-Interference	Explanation
Motivational variables	Internal PLOC External PLOC Introjected PLOC	Motivation-related variables, especially those stemming from own interests, advice, and shame, affect an individual’s adoption of the ePA.	Consistent with qualitative findings.	Individual motivation stemming from external mandates or internal feelings positively affects ePA adoption, although internal ones are stronger. In a conflict between external incentives and internal feelings of autonomous individuals, patients act in more protective ways and reject ePA usage.	Motivation has consistently been highlighted to be a strong predictor of adopting a wide range of technologies (e.g., [31,39]). Additionally, the sensitive nature of health information and resulting social pressures (i.e., shame) indicate rejection outcomes.
Self Efficacy	Mobile IT Identity	IT usage is motivated by a positive self-identification with IT use, and thus ePA adoption is.	IT identity was not significant.	A positive self-identification with IT has no direct effect on ePA adoption.	Even though the ePA is accessed through mobile applications, they do not require a self-identity attributed to “IT identity”.
[c]HIPC/Personal					
Characteristics	Age	Higher age results in deeper privacy concerns and lower ePA adoption.	Lower age results in deeper privacy concern.	Younger individuals express more privacy concern from using an ePA.	Demographics, such as age, are commonly associated with privacy concerns. Younger individuals may express more privacy concern attributed to their privacy literacy [48].
	Health Status	The health status negatively affects adoption stemming from the uneasiness of one’s severe health status.	Health status was not significant.	The self-perceived health status has no direct effect on the HIPC of ePA usage.	Statistic significance might fail to appear due to the low share of subjects with severe health status in our sample.
[c]HIPC/					
Perceptions	Risk	Perceived risk in processing by physicians and health insurance positively affects HIPC of using the ePA.	Consistent with qualitative findings.	Perceived risk add to the HIPC of using the ePA; however, trust in the physician or reasonable satisfaction with one’s health insurance lower privacy concerns.	Trust & risk are linked to privacy concerns [1,66,131]. Trust in physicians and the ePA lower privacy concerns [1,114].
	Trust	Trust in physicians or one’s health insurance outweigh perceived risks.			
	Information Sensitivity	Health information, when considered being sensitive, increases privacy concern.	Consistent with qualitative findings.	Individuals rate sensitivity of certain health information differently (i.e., towards STD), thus willing to share those data differs. Health information sensitivity is generally high.	Perceived sensitivity affects privacy concerns and intentions to provide health information [2]. Information sensitivity is associated with perceived risk [56].
HIPC	HIPC 3rd order formative	The interviews gave evidence for all constructs in the HIPC but *awareness*. In particular, the desire for control and granular permission management is strong, and the lack of those features hinders usage intentions.	Consistent with qualitative findings.	The HIPC significantly hinders ePA adoption intentions. However, the overall privacy concern is generally low in our sample.	Exercise of control over one’s health data is found essential. Granular permissions are often requested [27]. However, the privacy calculus is less profound where PHRs are relatively new. That is why individuals tend to weigh the benefits of the ePA more heavily than the concerns of privacy.

## Data Availability

The survey data are available in the Supplementary Materials to enable reproduction of the study’s results.

## Supplementary Materials

The survey data are available in https://www.mdpi.com/article/10.3390/ijerph18189553/s1.

## Appendix A

**Table A1:** Design Decisions for the Mixed-Methods-Design (Adapted from [46]).

	Property	Decision Consideration	Other Design Decision(s) Likely to Affect Current Decision	Design Decision and Reference to the Decision Tree
**Step 1:** decide on the appropriateness of mixed-methods research	Research questions	Qualitative or quantitative method alone was not adequate for addressing the research question. Thus, we used a mixed-methods research approach.	None	Identify the research questions We wrote the qualitative and quantitative research questions separately first, and a mixed-methods research question second. The qualitative research question was: “What are the salient factors determining an individual’s intentions toward using the ePA?” The quantitative research question was: “Does the research model explain usage intentions of the ePA?” The mixed-methods research question was: “Are the factors identified in the qualitative study and as captured through the research model supported by the results of the quantitative study?” We wrote the research questions in the question format. The quantitative research question depended on the results of the qualitative research question. The mixed-methods question depended on the results of both qualitative and quantitative research questions. The relationship between the questions and the research process is predetermined.
	Purposes of mixed-methods research	Mixed-methods research helps seeking convergence of results from different methods. We used mixed-methods research to develop hypotheses for empirical testing using the results of the qualitative.	Research questions	Developmental approach: mixed-methods with the findings from one method used to help inform the other method.
	Epistemological perspective	The qualitative and quantitative components of the study used different paradigmatic assumptions.	Research questions, purposes of mixed methods	Multiple paradigm stance.
	Paradigmatic assumptions	The researcher believed in the importance of research questions and embraced various methodological approaches from different worldviews.	Research questions, purposes of mixed methods.	Dialectic stance (an interpretive and grounded-theory perspective in the qualitative study and a positivist perspective in the quantitative study).
**Step 2:** develop strategies for mixed-methods research designs	Design investigation strategy	The mixed-methods study was aimed to develop and test a theory.	Research questions, paradigmatic assumptions	Study 1: exploratory investigation. Study 2: confirmatory investigation.
	Strands/phases of research	The study involved multiple phases.	Purposes of mixed-methods research	Multistrand design.
	Mixing strategy	The qualitative and quantitative components of the study were mixed at the data-analysis and inferential stages.	Purposes of mixed-methods research, strands/phases of research	Partially mixed methods.
	Time orientation	We started with the qualitative phase, followed by the quantitative phase.	Research questions, strands/phases of research	Sequential (exploratory) design.
	Priority of methodological approach	The qualitative and quantitative components were not equally important.	Research questions, strands of research	Dominant-less dominant design with the quantitative study being the more dominant paradigm.
**Step 3:** develop strategies for collecting and analyzing mixed-methods data	Sampling design strategies	The samples for the quant. & qual. components of the study differed but came from the same underlying population.	Design investigation strategy, time orientation	Purposive sampling for the qualitative study, probability sampling for the quantitative study.
	Data collection strategies	Qualitative data collection in phase 1. Quantitative data collection in phase 2.	Sampling design strategies, time orientation, phases of research	Qualitative study: closed- and open-ended questions with pre-designed interview guideline. Quantitative study: closed-ended questioning (i.e., traditional survey design).
	Data analysis strategy	We analyzed the qualitative data by finding broader categories using the software Atlas.ti. We analyzed the qualitative data first and the quantitative data second.	Time orientation, data collection strategy, strands of research	Sequential qualitative-quantitative analysis.
**Step 4:** draw meta-inferences from mixed-methods results	Types of reasoning	In our analysis, we focused on developing and then testing/confirming hypotheses.	Design-investigation strategy	Inductive and deductive theoretical reasoning.
**Step 5:** assess the quality of meta-inferences	Inference quality	The qualitative inferences met the appropriate qualitative standards. The quantitative inferences met the appropriate quantitative standards. We assessed the quality of meta-inferences.	Mostly primary design strategies, sampling-design strategies, data-collection strategies, data-analysis strategies, type of reasoning	We used conventional qualitative and quantitative standards to ensure the quality of our inferences. Design and explanatory quality; sample integration; inside-outside legitimation; multiple validities.
**Step 6:** discuss potential threats and remedies	Inference quality	We discussed all potential threats to inference quality in the form of limitations.	Data-collection strategies, data-analysis strategies	Threats to sample integration; sequential legitimation

## Appendix B. Interview Guideline

1. How would you describe your own privacy, especially on the Internet?
2. Has your information ever been used in an inappropriate manner?
3. Has your health information ever been used in an inappropriate manner?
  - How did you react/have you reacted?
4. How important is the smartphone in your life?
5. Are you currently using, or have you ever used any of these M-Health technologies?
  - Users: What technologies? What data? benefits? reasons for use?
  - Former users: Which technologies? Which data. Any advantages? Reasons for stopping use?
  - Non-users without experience: Would you ever use these technologies? What, why, perceived benefits.
6. Do you believe that you can improve your health through your own behavior?
7. Do you use a personal health record on your cell phone?
  - Can you tell us something about your experience with the app

*Presentation of the prototype (Figure 3)*

8. Which aspects of an ePA do you like? Which do you not?
9. What reasons would play a role in using the electronic patient file and the app?
  - What role does your interest in technology play?
  - What role do health factors play?
  - What role does the publisher of the app play?
10. Can you imagine your doctor prescribing via an app in the future?
  - What are the advantages?
11. What are your current concerns regarding the ePA app?
12. How would you describe your concerns about protecting your health data?
13. Which groups should have access to your health data, in your opinion?
14. Is it important for you to know how health data are used and shared?
15. Do you think that you currently have control over your health data?
16. How much control over your health data would you like to have?
17. Is it important for you to be able to restrict which individual documents an individual doctor can access?
18. When the ePA is introduced, would you give permission for your health data to be recorded?
19. How would you use the ePA app?
20. Do you believe that sharing data with physicians/therapists is associated with risks or negative consequences? (Why/what risks?)
21. What would you do if the app was mandatory on your smartphone tomorrow?

## Appendix C

**Table A2:** Interviewees from Qualitative Study.

#	Profile	Age	Insurance Status	Prior PHR Experience	Prior Privacy Invasion	Adoption Intention
I1	Student (IT related)	18–29	Statutory	No	No	Yes
I2	Public employee	30–49	Private	No	Yes	No
I3	Student (business related)	18–29	Statutory	No	No	Yes
I4	Retiree	50–69	Statutory	No	Yes	Yes

## Appendix D

**Table A3:** Emergent Themes from the Interviews.

Broader Category of Variables	Emergent Variable	I1	I2	I3	I4
Attitude	Attitude	✓	✓	✓	✓
Perceived Usefulness	Perceived Usefulness	✓	✓	✓	✓
Privacy Sensitivity	Privacy Sensitivity	✓			
Privacy Sensitivity	Privacy Risk Awareness	✓		✓	
PLOC	Interest in accessing data through own person	✓		✓	✓
PLOC	Likes to have full-fledged health manager	✓		✓	
PLOC	Likes to have sovereignty over data	✓			
PLOC	Interest in efficient treatments	✓	✓	✓	
PLOC	Shame		✓	✓	✓
PLOC	Political pressure		✓		✓
Health Status	Medical history/Health Status	✓	✓	✓	✓
Demographics	Age			✓	✓
Mobile IT identity	Dependence	✓	✓	✓	✓
IT experience	M-Health-Experience	✓	✓		
IT experience	IT experience			✓	✓
Inherent innovativeness	Interest in new innovations			✓	
Health Belief	Health Belief/Self-Efficacy	✓	✓	✓	
Prior privacy invasion	Experience		✓		✓
Prior privacy invasion	Response				✓
Information sensitivity	Overall perception of sensitivity	✓	✓		
Information sensitivity	Sensitive data types	✓	✓		
HIPC	General HIPC	✓	✓	✓	✓
HIPC	Desire for Privacy	✓		✓	✓
HIPC	Collection	✓			
HIPC	Secondary use	✓			
HIPC	Improper access	✓	✓	✓	✓
HIPC	Errors	✓			
HIPC	Control	✓	✓	✓	✓
HIPC	Awareness				
Perceived Ownership	Perception of Ownership	✓	✓		
Legislation awareness	Legislation awareness	✓			
Trust [health institution]	Trust			✓	✓
Trust [health professionals]	Trust				✓
Trust [technology vendors]	Trust	✓		✓	
Risk perception [health institution]	Risk perception	✓			
Risk perception [health professionals]	Risk perception	✓	✓		✓
Risk perception [technology vendors]	Risk perception			✓	
Usability	Usability	✓		✓	✓

## Appendix E

**Table A4:** Selected Quotes from the Interviews.

Category/Variable	Selected Quotes
Attitude	“I like the fact that all health information is stored in a digital file” (I1) “Well, I think the idea of centralization is key; I think it’s cool”. (I3)
Inherent innovativeness	“People who are critical about technology and digitization will not be able to do much with it and will not want to use it”. (I3)
Privacy sensitivity	“My concern is to ensure that as few companies as possible have access to my data”. (I1)
Mobile IT identity	“You don’t feel good if you don’t have [your smartphone] with you. Additionally, that’s kind of a weird feeling”. (I2)
Health Belief	“I am of the opinion that my own behavior has a serious influence on my own health”. (I1)
Internal PLOC	“I like the fact that all health information about the patient can be stored in a digital file, and the patient can, in theory, guarantee access to any doctor, any pharmacy, wherever necessary”. (I1) “I like the thought of seeing which current diagnoses I’m going to make or which doctor’s letters or whatever documents come together that exist about me”. (I1) “I have moved several times in my life now, even longer distances. Additionally, in the end, I always had to have everything handed over to me in physical form by the family doctor I was seeing”. (I3)
Introjected PLOC	“I think if you are seriously ill and you carry this app around with you all the time, it’s like carrying your X-rays around with you all the time. I don’t like the idea”. (I2) “Additionally, if someone is still in employment, and then have had a psychological rehab- I don’t know if everyone wants you to read that”. (I4)
Health Status	“People with serious chronic illnesses, psychological problems, that is, those who fall under social taboo topics will hardly use the app”. (I3)
HIPC Desire for Privacy	“I would feel safer now if the health insurance companies simply had access to what they now have in analog form”. (I1)
HIPC Control	“I’d like to decide for myself what the doctor can get from me, what insight he can get from me”. (I4)
HIPC Errors	“I can look at the file, [In case of errors] and I could check it. I could do something about it”. (I4)
HIPC Collection	“I know that many people are afraid that their contributions will increase as a result, or something similar”. (I1)
HIPC Improper Access	“Yes, the protocol is reasonably important. As I don’t want anyone to have someone who is [looking through documents] all the time when I give access to someone, although, of course, it could happen in my family doctor’s office that the trainee can read through everything, I will never notice”. (I2) “You can only open the ePA app when the phone is unlocked. Nevertheless, I find that these very sensitive personal data are very close to me, so that somebody might look into them”. (I2)
Information Sensitivity	“If it says in your documents, you have some sort of sexually transmitted disease or something; you may not want everyone to access it because it’s something that’s only your business”. (I2)
Perceived Ownership	“For me personally, it should be mainly the doctor who should be able to interact with this file”. (I1) “Do I wish control over it myself when my family doctor has the data? I would actually like to have confidence that the control will be realized by someone else”. (I2)
Risk Perception (Health professionals)	“Personally, I don’t think I would have a problem if my pharmacy knew what my medical history is”. (I1) “So currently, I have no worries because they are in a drawer or with some doctor. I’m not worried about that; I don’t want to. However, I’ll just assume that the doctors are abiding by the obligation of confidentiality”. (I3)
Risk perception (tech. vendors)	“I would personally reconsider my decision if the provider of the operating system, i.e., Apple or Google, would have access to my data”. (I3)
Trust (Health Professionals)	“I have confidence in the doctors where I have been. When I notice that the doctor is unpleasant, I go there only once, and then he will not see me again”. (I4) “I am still very unsure about these media, so I may not trust the media, unlike the doctors I go to”. (I4)
Trust (institution)	“I trust the health insurance companies; that plays an important role for me”. (I1) “I would feel more comfortable if there was an app from my own health insurance company, who would also take responsibility for it. That’s like in banking; it’s just a matter of trust”. (I3) “I am personally very, very satisfied with my health insurance company over the years. I am sure that it works well, and I can download the app with confidence. With third-party providers, I would have to deal with who is behind the app”. (I3)
Trust (technology vendors)	“If the app is supported by my health insurance company and is serious on a certain governmental, institutional level, then I would use the app. If any new third-party provider were to come around the corner, probably not”. (I3)

## Appendix F

**Table A5:** Scale Items for Construct Measures.

Name	Item	Mean	Std.dev.
**Intention (cf. [30,119])**			
Int1	I can imagine using the ePA app regularly.	3.840	1.281
Int2	I plan to use the ePA app in the future.	3.606	1.202
**External PLOC**			
I can imagine using the app*…*			
EPLOC1	*…*because my health insurance recommends it.	3.651	1.156
EPLOC2	*…*because it is recommended by my family doctor or other health professionals.	3.913	1.148
**Internal PLOC**			
I can imagine using the app*…*			
*Identified PLOC:*			
IPLOC1	*…*because I am interested in accessing my health data.	4.108	1.302
IPLOC2	*…*because I personally like using the app.	3.580	1.242
IPLOC3	*…*because I think it is important to me.	3.623	1.141
IPLOC4	*…*because I want to share my health data with other health professionals.	3.977	1.166
IPLOC5	*…*because I think it will result in more efficient treatments.	4.059	1.259
IPLOC6	*…*because I like to have sovereignty over my data.	3.863	1.206
IPLOC7	*…*because I would like to have all my health data in one central place.	4.068	1.279
*Intrinsic PLOC:*			
IPLOC8	*…*because I enjoy using an ePA.	3.517	1.094
**Introjected PLOC**			
IJPLOC1	I would feel bad if I didn’t use the ePA app. ${}^{a}$	2.204	1.145
IJPLOC2	I would use the ePA app because people I care about think I should use the app. ${}^{b}$	-	-
IJPLOC3	I feel political pressure from the government to use the app.	1.848	1.288
IJPLOC4	I find sharing my patient records and having constant access to my health history burdensome.	2.231	1.265
**Mobile Technology Identity [84,86]**			
Thinking about myself in relation to a mobile device, *…*			
*Dependence:*			
ITDep1	*…* I feel dependent on the mobile device.	3.027	1.168
ITDep2	*…* I feel needing the device.	3.505	1.030
*Emotional Energy:*			
ITEmo1	*…* I feel enthusiastic about the device.	3.680	0.867
ITEmo2	*…* I feel confident	4.312	1.239
**Health information privacy concern [15,69]**			
SUse1	I am concerned that my health information may be used for other purposes.	3.518	1.275
SUse2	I am concerned that my health information will be sold to other entities or companies.	3.376	1.232
SUse3	I am concerned that my health information will be shared with other entities without my authorization.	3.507	0.788
Control1	It is important to me that I have control over the health data I provide through the app.	4.532	0.665
Control2	It is important to me that I have control over how my health information is used or shared.	4.633	1.244
Control3	I fear a loss of control if my health data is available through the ePA app. ${}^{c}$	2.977	1.149
Errors1	I am concerned that my data in the ePA app may be incorrect.	2.792	1.145
Errors2	I am concerned that there is no assurance that my health information in the ePA app is accurate.	2.870	1.264
Errors3	I am concerned that any errors in my health data cannot be corrected.	2.811	1.241
Access1	I am concerned that my health data in the app is not protected from unauthorized access.	3.550	1.197
Access2	I am concerned that unauthorized persons may gain access to my health data.	3.639	1.249
Access3	I am concerned that there are insufficient security measures in place to ensure that unauthorized persons do not have access to my health data.	3.516	0.915
**Health status (cf. [14])**			
HStat1	I experience major pains and discomfort for extended periods of time.	1.576	0.886
HStat2	I believe that my general health is poor.	1.650	0.845
**Risk perceptions (cf. [15,53,69])**			
RiskHP1	It would be risky to disclose my personal health information to health professionals.	1.918	0.926
RiskHP2	There would be too much uncertainty associated with giving my personal health information to health professionals.	1.991	1.226
RiskIn1	It would be risky to disclose my personal health information to my health insurance.	2.512	1.240
RiskIn2	There would be too much uncertainty associated with giving my personal health information to my health insurance.	2.598	0.973
**Trust perceptions [15,53,69])**			
TrustHP1	I know health professionals are always honest when it comes to using my health information.	3.505	0.798
TrustHP2	I know health professionals care about patients.	3.782	0.797
TrustHP3	I know health professionals are competent and effective in providing their services.	3.696	0.843
TrustHP4	I trust that health professionals keep my best interests in mind when dealing with my health information.	3.742	0.978
TrustIn1	I know my health insurance is always honest when it comes to using my health information.	3.194	0.943
TrustIn2	I know my health insurance cares about customers.	3.395	0.973
TrustIn3	I know my health insurance is competent and effective in providing their services.	3.463	1.053
TrustIn4	I trust that my health insurance keeps my best interests in mind when dealing with my health information.	3.250	1.226
**Information sensitivity [70]**			
*Prompt: For each type of health information, choose the number that indicates how sensitive you feel this information is.*			
InfoSen1	Current health status	3.581	1.248
InfoSen2	Test results	3.764	1.287
InfoSen3	Health history	3.780	1.351
InfoSen4	Mental health	3.986	1.350
InfoSen5	Sexual health	3.854	1.381
InfoSen6	Genetic information	3.800	1.460
InfoSen7	Addiction information	3.712	0.806
**Demographics/Controls**			
Age	I am:		
	(1 = 18–24, 2 = 25–39, 3 = 40–59, 4 = 60+)		
Employment	What describes your employment status best?		
	(1 = Student, 2 = Retired, 3 = Employed, 4 = Other)		
Education	What is the highest level of education you have completed to date?		
	(1 = School, 2 = Abitur, 3 = Bachelor’s, 4 = Master’s/Diploma and above, 5 = N/A)		
M-health	Do you have experience using Health Apps or Smartwatches for Sport?		
	(1 = No Experience, 2 = Experience)		
HInsurance	Are you privately or statutorily insured?		
	(1 = Statutory, 2 = Private)		
**Data Quality [120]**			
Consent	I hereby confirm that I am at least 18 years old and that I have read and understood the declaration of consent and that I am a permanent resident of Germany.		
	(1 = No, 2 = Yes)		
DQRelunc	Now let’s be honest: Did you enjoy participating in this study?		
	(1 = No, 2 = Rather no, 3 = Rather yes, 4 = Yes)		
DQMeaningless	Did you perform all tasks as asked in each instruction?		
	(1 = I completed all tasks as required by the instructions, 2 = *Sometimes* I clicked something because I was unmotivated or just didn’t know my way around, 3 = I *frequently* clicked on something so I could finish quickly)		

^*a*^: Dropped after preliminary analysis. ^*b*^: Dropped after pilot study. ^*c*^: Dropped after preliminary analysis.

## Appendix G

**Table A6:** Distribution of Sample and German Citizens.

Dimension	Subgroup	Distribution		
		Sample		Germany
		Absolute	Share in %	Share in %
Age [in years]	18–24	19	9%	9%
	25–39	99	44%	23%
	40–59	79	36%	34%
	60+	25	11%	34%
Health insurance	Statutory Health Insurance	177	81%	87%
	Private Health Insurance	44	19%	11%
Education	With Graduation	47	21%	
	Abitur	55	25%	
	Bachelor’s degree	46	21%	
	Master’s degree/diploma or above	72	32%	
	Other	2	1%	
Employment	Student	25	11%	
	Retired	12	5%	
	Employed	133	60%	
	Other	52	24%	
Prior M-Health Experience	Is Adopter of Wearables or M-Health Technology	137	62%	
	No Adopter	85	38%	

## Appendix H

**Table A7:** Reliabilities of Multi-Item Constructs.

	Cronbach’s Alpha	Composite Reliability	Average Variance Extracted (AVE)
Access	0.972	0.982	0.947
Control	0.801	0.907	0.830
EPLOC	0.849	0.930	0.869
Errors	0.927	0.954	0.873
HealthStatus	0.824	0.917	0.847
IJPLOC	0.670	0.846	0.736
IPLOC	0.944	0.953	0.718
IT Dep.	0.788	0.904	0.825
IT Emo.	0.629	0.842	0.728
InfoSensitivity	0.950	0.959	0.770
Intention	0.920	0.962	0.926
RiskHP	0.922	0.962	0.927
RiskIn	0.963	0.982	0.964
SUse	0.952	0.969	0.913
TrustHP	0.878	0.915	0.730
TrustIn	0.911	0.937	0.789

## Appendix I

**Table A8:** Loadings of the Multi-Item Constructs.

	Loading	T Statistics	*p*-Value
Access1 ← Access	0.977	228.766	0.000
Access2 ← Access	0.976	232.272	0.000
Access3 ← Access	0.966	121.777	0.000
Control1 ← Control	0.878	12.868	0.000
Control2 ← Control	0.943	79.645	0.000
Control3 *(dropped from scale)*	-	-	-
EPLOC1 ← EPLOC	0.933	83.240	0.000
EPLOC2 ← EPLOC	0.931	57.362	0.000
Errors1 ← Errors	0.945	86.654	0.000
Errors2 ← Errors	0.957	115.480	0.000
Errors3 ← Errors	0.901	49.411	0.000
HealthStat1 ← HealthStatus	0.893	3.013	0.003
HealthStat2 ← HealthStatus	0.947	3.934	0.000
IJPLOC1 *(dropped from scale)*	-	-	-
IJPLOC2 *(dropped from scale)*	-	-	-
IJPLOC3 ← IJPLOC	0.762	10.887	0.000
IJPLOC4 ← IJPLOC	0.943	52.753	0.000
IPLOC1 ← IPLOC	0.872	42.133	0.000
IPLOC2 ← IPLOC	0.878	55.986	0.000
IPLOC3 ← IPLOC	0.870	51.110	0.000
IPLOC4 ← IPLOC	0.852	31.403	0.000
IPLOC5 ← IPLOC	0.844	32.052	0.000
IPLOC6 ← IPLOC	0.773	20.142	0.000
IPLOC7 ← IPLOC	0.851	32.225	0.000
IPLOC8 ← IPLOC	0.836	28.394	0.000
ITDep1 ← IT Dependency	0.900	50.240	0.000
ITDep2 ← IT Dependency	0.917	83.524	0.000
ITEmo1 ← IT Emo	0.879	42.837	0.000
ITEmo2 ← IT Emo	0.827	23.007	0.000
InfoSen1 ← InfoSensitivity	0.886	53.566	0.000
InfoSen2 ← InfoSensitivity	0.867	44.918	0.000
InfoSen3 ← InfoSensitivity	0.870	38.467	0.000
InfoSen4 ← InfoSensitivity	0.860	33.436	0.000
InfoSen5 ← InfoSensitivity	0.890	47.105	0.000
InfoSen6 ← InfoSensitivity	0.891	51.283	0.000
InfoSen7 ← InfoSensitivity	0.878	42.569	0.000
Int1 ← Intention	0.964	143.168	0.000
Int2 ← Intention	0.961	113.566	0.000
RiskHP1 ← RiskHP	0.957	94.154	0.000
RiskHP2 ← RiskHP	0.969	171.948	0.000
RiskIn1 ← RiskIn	0.982	196.133	0.000
RiskIn2 ← RiskIn	0.982	221.358	0.000
SUse1 ← SUse	0.948	102.306	0.000
SUse2 ← SUse	0.952	89.689	0.000
SUse3 ← SUse	0.966	142.763	0.000
TrustHP1 ← TrustHP	0.868	3.451	0.001
TrustHP2 ← TrustHP	0.861	3.550	0.000
TrustHP3 ← TrustHP	0.818	3.512	0.000
TrustHP4 ← TrustHP	0.869	3.455	0.001
TrustIn1 ← TrustIn	0.886	2.384	0.017
TrustIn2 ← TrustIn	0.902	2.392	0.017
TrustIn3 ← TrustIn	0.859	2.407	0.016
TrustIn4 ← TrustIn	0.905	2.388	0.017

## Appendix J

**Table A9:** Fornell–Larcker Criterion for Discriminant Validity of Multi-Item Constructs.

	1	2	3	4	5	6	7	8	9	10	11	12	13	14	15	16
**Access**	0.973															
**Control**	0.312	0.911														
**EPLOC**	−0.501	−0.081	0.932													
**Errors**	0.599	0.211	−0.458	0.934												
**HealthStat**	0.114	0.015	0.073	0.127	0.920											
**IJPLOC**	0.384	0.056	−0.443	0.333	0.092	0.858										
**IPLOC**	−0.494	−0.021	0.813	−0.468	0.101	−0.481	0.848									
**ITDep**	−0.135	−0.137	0.288	−0.176	0.075	0.006	0.210	0.908								
**ITEmo**	−0.236	−0.109	0.302	−0.254	−0.15	−0.181	0.216	0.353	0.853							
**InfoSen**	0.120	0.142	−0.261	0.118	−0.002	0.118	−0.211	−0.209	−0.038	0.878						
**Intention**	−0.555	−0.08	0.801	−0.472	0.057	−0.503	0.846	0.215	0.242	−0.256	0.962					
**RiskHP**	0.317	−0.009	−0.408	0.353	0.214	0.382	−0.363	-0.029	−0.171	0.132	−0.385	0.963				
**RiskIn**	0.336	0.142	−0.29	0.298	0.138	0.241	−0.295	−0.019	−0.145	0.216	−0.3	0.394	0.982			
**SUse**	0.824	0.292	−0.528	0.552	0.107	0.392	−0.542	−0.105	−0.202	0.242	−0.576	0.353	0.404	0.955		
**TrustHP**	−0.224	0.001	0.384	−0.175	−0.031	−0.287	0.235	0.136	0.314	−0.041	0.251	−0.332	−0.139	−0.226	0.854	
**TrustIn**	−0.297	−0.09	0.427	−0.274	0.033	−0.238	0.389	0.154	0.176	−0.136	0.338	−0.247	−0.424	−0.366	0.447	0.888

Diagonal numbers represent the square-root of the AVEs. Each SQAVE exceeds all correlations with other latent constructs.
